# Integrating data from asymmetric multi-models can identify drought-resistant groundnut genotypes for drought hot-spot locations

**DOI:** 10.1038/s41598-023-38581-0

**Published:** 2023-08-05

**Authors:** B. C. Ajay, Narendra Kumar, Praveen Kona, K. Gangadhar, Kirti Rani, G. A. Rajanna, S. K. Bera

**Affiliations:** ICAR-Directorate of Groundnut Research, Junagadh, 362001 Gujarat India

**Keywords:** Plant sciences, Environmental social sciences

## Abstract

Water/drought stress experiments are frequently conducted under imposed stress or rainout shelters, while natural drought hot-spot investigations are rare. The “drought hot spot” in Anantapur, Andhra Pradesh, India, is appropriate for drought stress evaluation due to its hot, arid environment, limited rainfall, with over 50% rainfall variability. According to reports, 30 out of 200 groundnut cultivars in India are supposed to possess drought-tolerant characteristics. However, these cultivars are yet to be evaluated in areas that are prone to drought. This study tested these drought-tolerant genotypes in naturally drought-prone areas of Anantapur under rainfed conditions from Kharif 2017 to 2019. Pod yield and rainfall-use-efficiency (RUE) were measured for these genotypes. Genotype and genotype*environment interactions affected pod yield and RUE (GEI). The AMMI model exhibits significant season-to-season variability within the same area with environmental vectors > 90° angles. GGE biplot suggested the 2018 wet season for drought-resistant cultivar identification. Kadiri5 and GPBD5 were the most drought-tolerant cultivars for cultivation in Anantapur and adjacent regions. These types could also be used to generate drought-tolerant groundnut variants for drought-prone regions.

## Introduction

Drought is the principal stress among abiotic factors causing significant crop loss in arid and semi-arid environments^1^. Groundnut is one of the most cherished oilseed crop grown in these regions, valued both as a seed crop and as a feed crop. Almost 85 percent of the groundnut area remains rainfed, with approximately 80% falling under dryland with no irrigation facilities^2^. Water stress in dryland crops is unexpected and sporadic^3^, resulting in considerable yield loss from pre-flowering to flowering, pegging, and pod formation^4^. Up to 88% of groundnut production was lost due to drought stress during pod-setting^5^. Under drought stress, not only yield but also product quality diminishes^6^. From peg initiation through pod filling, drought stress can significantly diminish pod output^1,7^.

Studies on drought stress evaluation is conducted under imposed water stress conditions either under rainout shelters or during post-rainy situations^8,9^. These stress situations rarely simulate actual drought situations being faced in drought prone regions with hot and arid climatic conditions. Such natural drought prone regions could be referred to as “Drought hot spots” and these hot spots face frequent dry spells and characterized with low and irregular rainfall distribution. The district of Anantapur in Andhra Pradesh, India, is more frequently susceptible to drought and is characterized by hot and arid climate with low and irregular rainfall distribution. 66 of the 133 years of record, between 1876–1877 and 2008–2009, were drought years^10,11^. Soils at Anantapur are red sandy loam (alfisols), are very shallow (0.1–0.3 m deep) and have compact sub-surface which restrict the root growth. Considering the rainfall patterns and soil characteristics, Anantapur is considered as hot-spot for drought. Studying genotypic performances in such drought hot-spot locations would help in identifying actual drought tolerant genotypes.

Groundnut is an important crop of Anantapur and adjoining regions. A cluster of districts consisting of Anantapur, Kurnool, and Chittoor in Andhra Pradesh and Bellary, Chickballapur, Chitradurga, and Tumkur in Karnataka (approximately 11–12.5 lakh hectares) account for nearly 25% (48.1 lakh ha) of the nation’s groundnut area (Table 1). Normal kharif rainfall (June–October) is approximately 500 milli metres, while rabi rainfall (November–February) is approximately 150 milli metres. Water scarcity has emerged as a significant constraint on groundnut production in this region, resulting in a 15–30% decline in productivity relative to the national average. Low yields followed by enormous year-to-year production shifts are primarily the result of insufficient and uneven rainfall distribution^10^. The influence of length of dry period at various growth stages of groundnut on pod yield at Anantapur revealed that yields decrease if the stress lasts for three weeks or longer during the early vegetative stage (0–35 DAS). The effect will be more pronounced at the beginning of pegging and seed development (51–85 DAS). Pod yields decreased dramatically in years where the dry season lasted more than 30 days during this stage. The recurrent failure of monsoon rains during these crucial times is at the heart of the district's agricultural failures^10^. The average productivity of groundnut in these districts for the period 2018–2019 to 2020–2021 is 841 kg/ha (Table 1), which is less than the national triannual average of 1720 kg/ha for the period 2018–2019 to 2020–2021^12^.

**Table 1:** Area, production and productivity of groundnut in Anantapur and adjoining districts during 2011–2012 to 2020–2021.

State	District	2011–2012	2012–2013	2013–2014	2014–2015	2015–2016	2016–2017	2017–2018	2018–2019	2019–2020	2020–2021	Min	Max	CV
Area (ha)														
Andhra Pradesh	Chittoor	132,056	135,000	143,027	122,578	114,394	133,124	115,048	99,275	95,746	123,000	95,746	143,027	12.72
	Kurnool	84,011	119,000	150,918	94,160	73,370	113,447	85,784	89,187	79,542	94,000	73,370	150,918	23.68
	Anantapur	733,960	709,000	711,145	550,794	444,657	608,162	402,435	474,392	371,029	481,000	371,029	733,960	24.59
Karnataka	Bellary	56,185	49,598	45,709	47,214	42,025	33,385	53,312	55,751	53,908	52,334	33,385	56,185	14.57
	Chikballapur	29,316	21,826	18,722	18,166	19,466	25,391	17,254	24,981	24,826	34,553	17,254	34,553	23.48
	Chitradurga	90,519	71,002	111,650	118,724	93,516	128,536	90,618	74,641	116,877	156,684	71,002	156,684	24.98
	Tumkur	87,050	82,878	71,042	86,127	63,437	92,135	53,872	63,528	48,641	85,134	48,641	92,135	20.93
	Total	1,213,097	1,188,304	1,252,213	1,037,763	850,865	1,134,180	818,323	881,755	790,569	1,026,705			17.12
Production (ton)														
Andhra Pradesh	Chittoor	82,799	87,000	142,455	72,811	130,867	71,887	214,449	89,645	165,832	127,000	71,887	214,449	39.39
	Kurnool	20,835	88,000	136,128	67,607	66,620	99,153	121,985	42,542	125,040	95,000	20,835	136,128	43.08
	Anantapur	174,682	296,000	286,591	148,714	319,708	140,485	412,093	150,857	277,530	228,000	140,485	412,093	37.03
Karnataka	Bellary	26,561	33,595	56,364	46,513	38,726	40,438	80,528	61,914	72,261	68,558	26,561	80,528	34.39
	Chikballapur	17,639	10,139	16,665	9405	16,902	8467	22,276	12,056	28,042	34,138	8467	34,138	48.09
	Chitradurga	43,119	41,415	79,975	77,034	60,411	26,986	93,577	30,349	80,166	133,338	26,986	133,338	49.42
	Tumkur	20,326	23,699	35,230	42,056	43,150	29,935	42,120	36,332	43,205	68,618	20,326	68,618	34.82
Productivity (kg/ha)														
Andhra Pradesh	Chittoor	627.00	644.44	996.00	594.00	1144.00	540.00	1864.00	903.00	1732.00	1036.00	540.00	1864.00	46.24
	Kurnool	248.00	739.50	902.00	718.00	908.00	874.00	1422.00	477.00	1572.00	1014.00	248.00	1572.00	44.43
	Anantapur	238.00	417.49	403.00	270.00	719.00	231.00	1024.00	318.00	748.00	473.00	231.00	1024.00	54.45
Karnataka	Bellary	472.74	677.35	1233.11	985.15	921.50	1211.26	1510.50	1110.55	1340.45	1310.00	472.74	1510.50	29.56
	Chikballapur	601.69	464.54	890.13	517.73	868.28	333.46	1291.06	482.61	1129.54	988.00	333.46	1291.06	42.46
	Chitradurga	476.35	583.29	716.30	648.85	646.00	209.95	1032.65	406.60	685.90	851.00	209.95	1032.65	36.68
	Tumkur	233.50	285.95	495.90	488.30	680.20	324.90	781.85	571.91	888.24	806.00	233.50	888.24	41.48

The development of water-stressed genotypes will be aided by an understanding of how well genotypes survive in arid environments. In general, genotypes selected for adaptability and performance under high input conditions are poorly suited to low input environments^13^. In India, more than 200 varieties have been released for commercial cultivation by a central or state varietal release committee^14^, of which 30–35 varieties have been reported to possess drought resistance but this has not been validated in drought hot spot locations.

Thus, the purpose of this study was to determine a variety’s potential to grow in Anantapur’s sparse rainfall conditions while maintaining steady yields throughout the seasons. Understanding how well genotypes survive in low-rainfall environments will benefit in the development of drought-tolerant genotypes. Notwithstanding fluctuations in annual weather patterns, these drought-resistant genotypes will be able to provide yields that are much greater over time. To investigate varietal stability and considerable year-to-year yield fluctuations use of stability models such as Additive main effect and multiplicative interaction (AMMI)^15–18^ and GGE biplots were utilised.

## Material and methods

### Experimental materials

The experimental material comprised of thirty cultivars that had been released for cultivation in different parts of India and were considered to be drought-tolerant^14^. Table 2 provides a list of these varieties, along with their release year and area of adoption. These materials were sourced from our own gene bank at ICAR-Directorate of Groundnut Research, Junagadh.

**Table 2:** List of groundnut varieties used in the study.

S. no	Variety	Year of release	Parentage	Area of adoption	Botanical group
1	ABHAYA	2007	K 134 × TAG 24	Andhra Pradesh	Spanish
2	AK 265	2007	ICGS 11 × US 63	Southern Maharashtra, AP, TN and Karnataka	Virginia Bunch
3	ANANTHA	2010	--	Andhra Pradeh	
4	CSMG 84-1	1992	Selection from MA10	Rajasthan, Uttar Pradesh and Haryana	Virginia Runner
5	Dh 3-30	1975	Spanish Improved x US4	Northern Karnataka	Spanish Bunch
6	DHARANI	2012	VRI -2 × TCGP – 6	Andhra Pradesh	
7	DRG 17	1994	Robout 33-1 × TAP5	Rajasthan, Punjab, Uttar Pradesh and Haryana	Virginia Bunch
8	DSG 1	1997	Selection from Mardur local	Karnataka	Spanish Bunch
9	GG 2	1983	J11 × EC 16659	Gujarat	Virginia Bunch
10	Girnar 2	2008	M13 × Robout 33–1	Uttar Pradesh, Punjab, North Rajasthan	Virginia Bunch
11	GPBD 5	2010	TG 49 × GPBD 4	Jharkhand and Manipur	Spanish
12	ICGS 1	1990	Selection from Robut 33-1	UP, Bihar, Haryana, Punjab and Rajasthan	Spanish
13	ICGS 44	1988	Selection from Natural hybrid population of Robut 33-1	Gujarat, became popular in AP, Karnataka, Orissa and TN	Spanish
14	ICGS 76	1989	TMV 10 × Chico	Southern Maharashtra and Karnataka	Virginia Bunch
15	ICGV 86031	1991	F334A-B-14 and NC Ac 2214	–	Spanish
16	ICGV 86325	1994	ICGS 20 × G 201	Southern Maharashtra, AP, TN and Karnataka	Virginia Bunch
17	ICGV 91114	2007	ICGV 86055 × ICGV 86533	Andhra Pradesh	Spanish
18	K 6	2005	JL 24 × Ah316/S	Andhra Pradesh	Spanish
19	K 9	2009	Kadiri 4 × K 134	Andhra Pradesh	Spanish
20	KADIRI 5	2005	JL 24 × VG 55-7	Andhra Pradesh	Spanish
21	KADIRI HARITHANDRA	2010	91/57-2 × PI-476177	Karnataka and Maharashtra	
22	MUTANT 3 (Co-2)	1983	EMS mutant of Pollachi 1	Tamil Nadu	Spanish
23	R 2001-2	2010	ICGS 11 × ICG 4728	West Bengal, Orissa, Jharkhand, Southern Maharashtra, AP, TN and Karnataka	Spanish Bunch
24	R 2001-3	2008	ICGS 11 × ICG 4728	Southern Maharashtra, AP, TN and Karnataka	Spanish
25	R 8808	1997	ICGS 11 × Chico	Kaataka, Andhra Pradesh, Tamil Nadu	Spanish Bunch
26	SPANISH IMPROVED	1905	Selection from Spanish Peanut	Tract of Bombay and Karnataka	Spanish
27	TAG 24	1991	Selection from TGS 2 × TGE 1	Maharashtra	Spanish
28	TDG 39	2009	TAG 24 × TG 19	Karnataka	Virginia Bunch
29	TG 72	–	Mutant of TG 38	–	
30	TMV 2	1940	Selection from Gudhiyatham bunch	Tamil Nadu, Andhra Pradesh and Karnataka	Spanish

### Experimental locations and meteorological data

**Figure 1:**
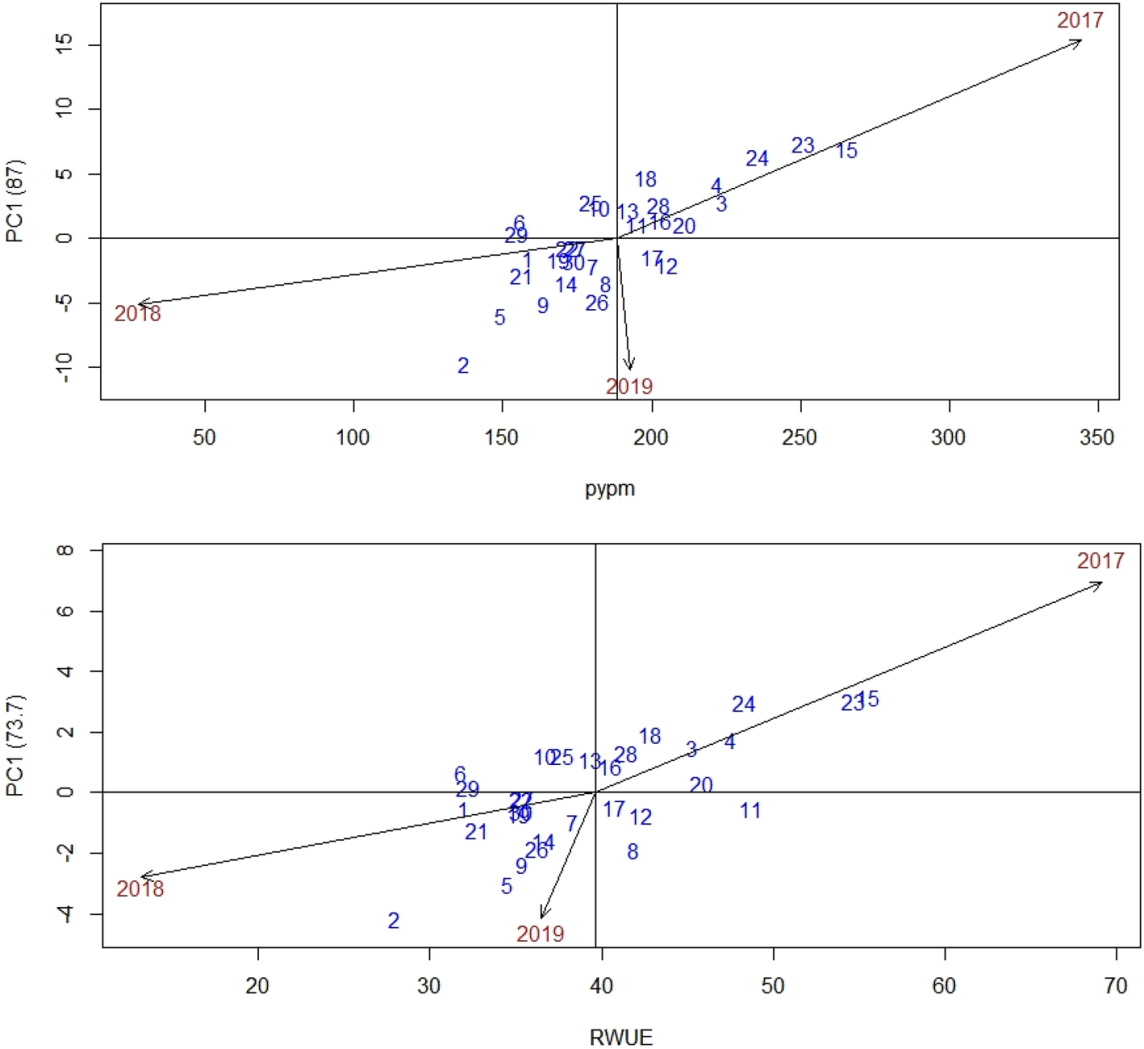
AMMI biplots of (**a**) PC1 vs Pod yield (PYPM) and (**b**) PC1 vs Rain water use efficiency (RUE). 1, Abhaya; 2, AK 265; 3, Anantha; 4, CSMG 84-1; 5, Dh 3-30; 6, Dharani; 7, DRG 17; 8, DSG 41; 9, GG 2; 10, Girnar 2; 11, GPBD 5; 12, ICGS 1; 13, ICGS 44; 14, ICGS 76; 15, ICGV 86031; 16, ICGV 86325; 17, ICGV 91114; 18, K6; 19, K9; 20, Kadiri 5; 21, Kadiri Haritandra; 22, Mutant 3; 23, R 2001-2; 24, R 2001-3; 25, R 8808; 26, Spanish Improved; 27, TAG 24; 28, TDG 39; 29, TG 72; 30, TMV 2.

Experiments were done at the Regional Research Station (RRS) of the ICAR-Directorate of Groundnut Research in Anantapur (latitude: 14° 41′ N, longitude: 77° 67′ E) during the three consecutive rainy seasons of 2017, 2018, and 2019. Red sandy loam of shallow depth, low in organic carbon (0.35%) and available nitrogen (142 kg/ha), medium in accessible phosphorus (32 kg/ha) and potassium (226 kg/ha) was the soil at the experimental site. The climate in the region of study is semi-arid, therefore it is hot and dry for the majority of the year, with average highs about 37 °C. The average weekly low temperature ranged from 19.70 °C in November to 25.30 °C in July, and the average weekly high temperature ranged from 35.4 °C in July to 35.70 °C in October. Although though the groundnut crop was grown in the same fixed pattern throughout the research years, the rainfall obtained in 2017, 2018, and 2019 varied from 504.1 to 228.0 to 538.2 mm, respectively (Fig. 1S). In 2018, moisture stress has become the most significant limiting factor in the study area.

### Experimental design and data collection

Seeds were sown directly in a randomised complete block design (RCBD) with two replications during the third week of July. Each genotype was planted in 3 m rows of single rows per genotype each replicate. Plant geometry of 30 cm row-to-row and 10 cm plant-to-plant distance was maintained in each plot. Crops were harvested when they reached maturity. After drying pods, pod yields were recorded on a plot-by-plot basis. According to Oweis^19^, Rainwater use efficiency (RUE) was computed by dividing groundnut pod yield by cumulative rainfall received from seeding to harvest as follows: Pod yield (g/m^2^)/Rainfall (mm).

RUE denotes the yield achieved by a genotype per milli metre of rain water received during the study period. Because the crop receives no irrigation other than rain water, RUE would also reflect a genotype's water productivity or water use efficiency under rainfed conditions.

### Statistical analysis

#### AMMI and GGE biplot analysis

AMMI stability model calculates environment and genotype main effect and, multiplicative effects of GEI. The AMMI stability analysis was performed using package ‘agricolae’^20^ in R^21^ and the model is represented as

$${\text{Y}}_{{{\text{ijk}}}} = \, \mu \, + {\text{G}}_{{\text{i}}} + {\text{ E}}_{{\text{i}}} + \, \sum \lambda_{{\text{k}}} \alpha_{{{\text{ik}}}} \gamma_{{{\text{jk}}}} + {\text{d}}_{{{\text{ij}}}} + {\text{e}}_{{{\text{ijk}}}}$$

where the response variable such as pod yield and RUE is represented by Y_ijk_, grand mean represented by μ, genotype deviation from μ represented by G_i_, environment deviation from μ represented by E_j_, eigen value of kth interactive principal component (IPCA) represented by ʎ_k_, IPCA score for ith genotype on kth IPCA represented by α_ik_, IPCA score of jth environment for kth IPCA represented by $\gamma_{{{\text{jk}}}}$, residual GEI unexplained by model represented by d_ij_ and model error represented by e_ijk_. The sum of square (SS) due to GEI signal^22^ was estimated as

$${\text{SS}}_{{{\text{Signal}}}} = {\text{ SS}}_{{({\text{GEI}})}} - {\text{ SS}}_{{{\text{Noise}}}}$$

$${\text{SS}}_{{{\text{Noise}}}} \, = \,{\text{degree}}\;{\text{ of}}\;{\text{ freedom}}_{{({\text{GEI}})}} \, \times \,{\text{Mean }}\;{\text{squares }}\;{\text{of }}\;{\text{residuals}}{.}$$

The stable performance of genotypes in AMMI stability model was calculated as Modified AMMI Stability Index (MASI)^23^ in R using the package ‘ammistability’^24,25^ as

$$MASI = \sqrt {\mathop \sum \limits_{n = 1}^{{N^{\prime}}} PC_{n}^{2} \times \theta_{n}^{2} }$$

PCn are the scores of nth IPC; and θn is the percentage sum of squares explained by the nth principal component interaction effect.

Simultaneous stability index (SSI) was used to compare stability of high pod yielding and RUE genotypes and ranking the genotypes by combining both yield and stability parameters^26^. The SSI for each genotype was estimated in R using the package ‘ammistability’^24,25^ as

$${\text{SSI }} = {\text{ rSP }} + {\text{ rY}}$$

where, rSP is the rank of MASI stability value and rY is the rank of adjusted mean pod yield and RUE of genotype across environments. GGE-biplot analysis was performed on pod yield separately for Spanish and Virginia groups using ‘GGEbiplotGUI’ package^34^ in R^21^.

### Handling plant materials

The collection and handling of plant were in accordance with all the relevant guidelines.

## Results

### AMMI stability analysis for Pod yield and RWUE

For pod yield and RUE, the AMMI ANOVA revealed the significance of the mean sum of squares due to drought environments, genotypes, and GEI (Table 3). The main effects of drought environment, genotype, and GEI accounted for 81.76, 4.29, and 12.80% of phenotypic variability in pod yield and 74.87, 6.18, and 17.50% of phenotypic variability in RUE, respectively. The variance caused by GEI was further subdivided into variance caused by signal and noise (Table 4). The variance caused by signal occurred due to known factors such as genotypes and phase of drought, whereas noise variation was attributed to mistake caused by unknown model factors^22^. In our experiments, pod yield and RUE recorded 94.29 and 94.76 percent of GEI owing to signal and the remainder due to noise, respectively. This indicates that the AMMI stability model is adequate for comprehending the GEI^27^. The multiplicative component of AMMI models consists of the singular value/multiplication factor of IPCA, the genotype eigenvector, and the environment eigenvector^17^. The IPCA1 and IPCA 2 were highly significant for pod yield and RUE, with IPCA1 accounting for 86.99 and 73.33 percent of the GEI for pod yield and RUE, respectively, whereas IPCA 2 accounted for 13.01 and 26.27 percent of the GEI, indicating a significant contribution of environment on genotype and trait expression performance.

**Table 3:** AMMI analysis of variance of 30 groundnut varieties evaluated at drought hot spot location for three rainy seasons for pod yield, and Rain Water use efficiency (RUE).

Source of variation	Df	Pod yield (g/m^2^)			Rain water use efficiency (g/m^2^/mm)		
		Sum Sq	Mean Sq	% SS	Sum Sq	Mean Sq	% SS
Environment (E)	2	3,009,690	1,504,845***	81.76	94,802	47,401***	74.87
Rep(E)	3	1712	571	0.05	68	23	0.05
Genotype (G)	29	158,000	5448***	4.29	7827	270***	6.18
G*E	58	471,146	8123***	12.80	22,152	382***	17.50
PC1	30	409,852	13,662***	86.99	16,332	544***	73.73
PC2	28	61,294	2189***	13.01	5819	209***	26.27
Residuals	87	40,359	464	1.10	1770	20	1.40

*,**,*** Significant at 5%, 1% and 0.1% level, respectively.

**Table 4:** Estimates of sum of square due to signals and noises by using AMMI model for drought stress in groundnut.

Traits	Sum of squares		Percent variation	
	GEI_signal_	GEI_noise_	GEI_signal_	GEI_noise_
Pod yield	444,234	26,912	94.29	5.71
RUE	20,992	1160	94.76	5.24

GEI genotype × environment interaction,

### AMMI 1 biplot

The biplot abscissa and ordinate reflected the 1st principal component (IPCA1) term and the trait's substantial significance in additive main effects and multiplicative interaction 1 (AMMI 1). Figure 1 depicts the additive main effects and multiplicative interaction effect of 30 genotypes on pod yield and RUE throughout three rainy seasons in this study. The rainy season of 2018 had a IPCA1 score or vector closer to zero for pod yield (Fig. 1a) and RUE (Fig. 1b) compared to prior rainy seasons, showing a reduced interaction effect, which almost guaranteed the competitive advantage of genotypes in that season and considered suitable for genotype evaluation. Variety GPBD 5 (11) and TG 72 (29) received near-zero scores on the IPCA1 axis, indicating that it is less affected by the environment. PC 1 scores next to zero lines of biplot suggested that varieties ICGS 44, (13), GPBD 5 (11), and Girnar 2 (10), were suitable for all conditions. Varieties with PC1 vectors of the same sign and score but away from zero biplot lines suggested that they were suited to a specific environment. Variety ICGV 86031 (15) was discovered to be suited for the rainy season of 2017 and variety AK 265 (2) for the rainy season of 2018, as both genotype and environment exhibited the same sign. According to Murphy et al.^28^, Mogale et al.^29^, and Oladosu et al.^30^, when the IPCA1 score for a genotype or environment is close to zero, there is a small interaction impact; on the other hand, if a genotype and environment achieve the same sign on the PCA axis, there is a positive interaction; otherwise, there is a negative interaction.

### AMMI based stability indices for cultivar performance in groundnut

The variety with the lowest Modified AMMI stability Index (MASI) calculated from the IPCA axis and IPCA scores is the most stable^1,23,24^ (Table 5). Under drought stress, TG 72, TAG 24, and Mutant 3 had the lowest MASI for pod yield, with pod yields of 154.7, 174.4, and 171.6 g/m^2^ respectively. MASI values of 6.03, 6.38, and 5.53 were obtained for high pod yielding genotypes such as ICGV 86031, R 2001-2, and R 2001-3, respectively. Mutant 3, TG 72, and TAG 24 had the lowest MASI for RUE. The MASI for the high RUE genotypes ICGV 86031, R 2001-2, and GPBD 5 was 2.32, 2.24, and 1.41, respectively.

**Table 5:** Ranking of 30 groundnut varieties for pod yield, and rain water use efficiency (RUE).

	Pod yield (g/m^2^)					Rain water use efficiency (g/m^2^/mm)				
	MASI	rMASI	means	rY	SSI	MASI	rMASI	means	rY	SSI
ABHAYA	1.33	7	158.5	25	32	0.41	5	32.00	28	33
AK 265	8.46	30	137.1	30	60	3.09	30	28.00	30	60
ANANTHA	2.51	19	223.7	4	23	1.17	18	45.28	7	25
CSMG 84-1	3.67	22	221.9	5	27	1.31	20	47.56	5	25
Dh 3-30	5.21	26	149.5	29	55	2.28	28	34.52	25	53
DHARANI	1.14	5	155.9	27	32	0.48	6	31.83	29	35
DRG 17	1.87	13	180.4	17	30	0.73	11	38.33	15	26
DSG 41	3.06	21	185.1	14	35	1.45	22	41.89	10	32
GG 2	4.40	25	163.6	24	49	1.73	25	35.41	21	46
Girnar 2	2.04	15	182.1	15	30	0.90	14	36.72	17	31
GPBD 5	1.37	9	194.8	12	21	1.41	21	48.70	3	24
ICGS 1	1.81	12	204.9	7	19	0.65	10	42.28	9	19
ICGS 44	1.94	14	191.7	13	27	0.81	13	39.37	14	27
ICGS 76	3.03	20	171.2	22	42	1.18	19	36.59	18	37
ICGV 86031	6.03	28	265.4	1	29	2.32	29	55.48	1	30
ICGV 86325	1.24	6	202.7	8	14	0.76	12	40.49	13	25
ICGV 91114	1.35	8	200.0	10	18	0.51	7	40.75	12	19
K 6	4.11	23	198.0	11	34	1.46	23	42.78	8	31
K 9	1.43	10	168.7	23	33	0.51	9	35.15	24	33
KADIRI 5	0.91	4	211.0	6	10	0.28	4	45.86	6	10
KADIRI HARITHANDRA	2.46	18	156.6	26	44	0.91	16	32.80	26	42
MUTANT 3	0.64	3	171.6	21	24	0.19	1	35.29	23	24
R 2001-2	6.38	29	250.8	2	31	2.24	27	54.65	2	29
R 2001-3	5.53	27	235.5	3	30	2.20	26	48.29	4	30
R 8808	2.42	17	179.4	18	35	0.90	15	37.68	16	31
SPANISH IMPROVED	4.27	24	181.8	16	40	1.48	24	36.21	19	43
TAG 24	0.62	2	174.4	19	21	0.21	3	35.42	20	23
TDG 39	2.30	16	202.3	9	25	0.98	17	41.39	11	28
TG 72	0.47	1	154.7	28	29	0.21	2	32.25	27	29
TMV 2	1.54	11	173.9	20	31	0.51	8	35.31	22	30

Simultaneous selection indices (SSI) for genotypes were calculated using the sum of MASI ranks and genotype ranks determined from pod yield and RUE (Table 5). The genotypes with the lowest SSI values are the most stable and function well across environments^23–25,31^. Kadiri 5, ICGV 86325, ICGV 91114, and ICGS 1 had the lowest SSI for pod yield, indicating that these genotypes have the ability to combine high stability and high pod yield across environments. For RUE, Kadiri 5, ICGV 91114 and ICGS 1 had the lowest SSI, indicating that these genotypes had high stability and high RUE across environments. Kadiri 5 had the highest pod yield (211.0 g/m^2^) and high RUE (45.86 g/m^2^-mm) with low SSI values. As a result, in drought stress conditions, Kadiri 5 is regarded as the most stable drought tolerant high yielding variety.

### GGE biplot to visualize GEI for pod yield and RUE

**Figure 2:**
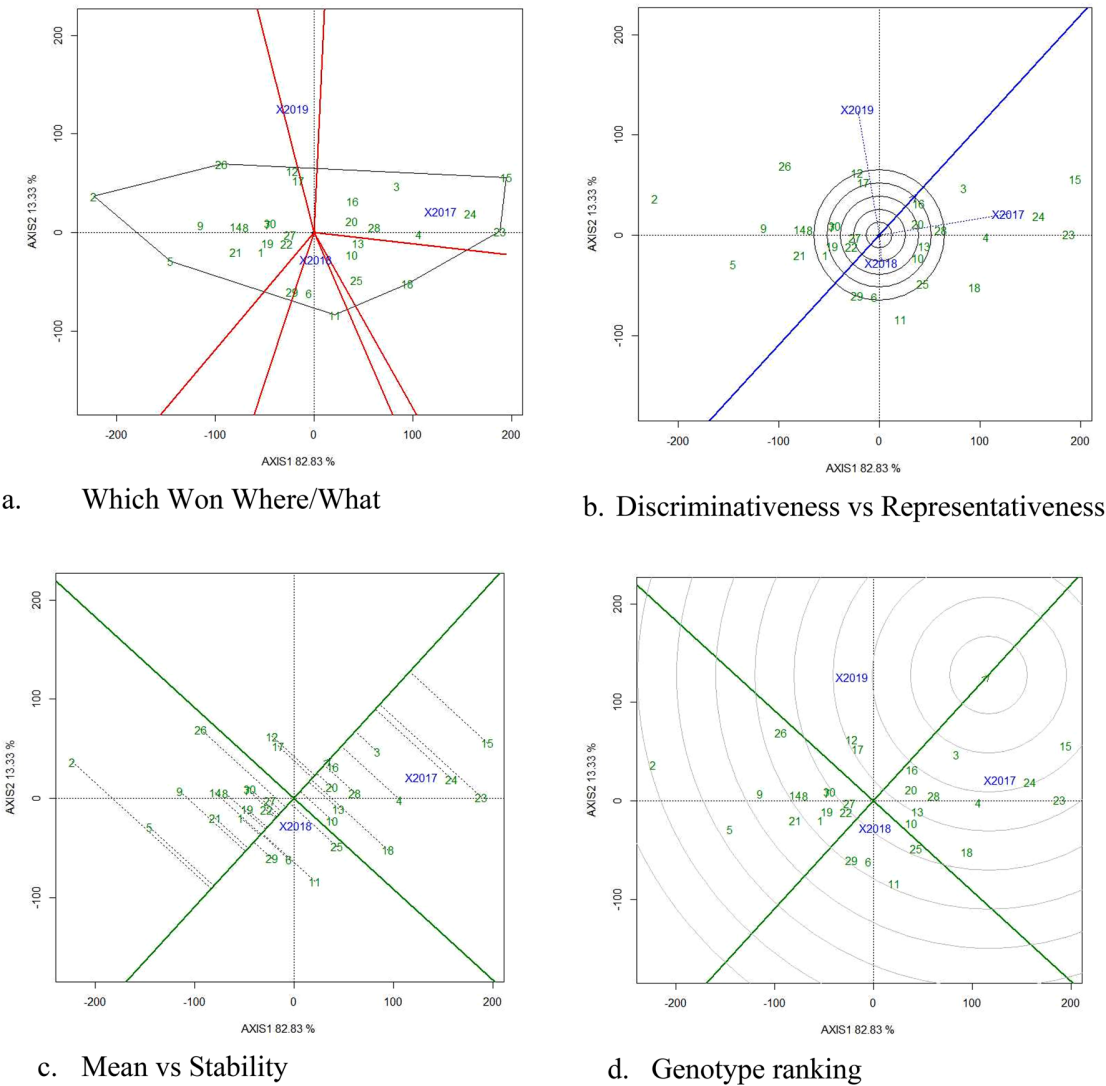
GGE Biplots of for pod yield (g/m^2^) under drought stress. The data is not transformed, not scaled, environments were centered (Centering = 2) and the biplots based on singular value partition by column metric preserving. The Biplots explained 79.86 percent of G + GE. (**a**) Which won where/what GGE biplot to visualise the mega environment and environment specific genotypes. (**b**) discriminativeness vs representativeness GGE biplot depicting the longest environment vector and nearer to average environmental coordinates and identified 2018 as highly discriminative environment, (**c**) mean vs stability GGE biplot depicting the 18 and 23 located in the direction oGGE biplotf the average environmental, (**d**) Ranking of genotype GGE biplot depicting the genotype 11 located in near to ideal genotype for pod yield (g/m^2^). 1, Abhaya; 2, AK 265; 3, Anantha; 4, CSMG 84-1; 5, Dh3-30; 6, Dharani; 7, DRG 17; 8, DSG 41; 9, GG 2; 10, Girnar 2; 11, GPBD 5; 12, ICGS 1; 13, ICGS 44; 14, ICGS 76; 15, ICGV 86031; 16, ICGV 86325; 17, ICGV 91114; 18, K 6; 19, K 9; 20, Kadiri 5; 21, Kadiri Hariandra; 22, Mutant 3; 23, R 2001-2; 24, R 2001-3; 25, R 8808; 26, Spanish Improved; 27, TAG 24; 28, TDG 39; 29, TG 72; 30, TMV 2.

**Figure 3:**
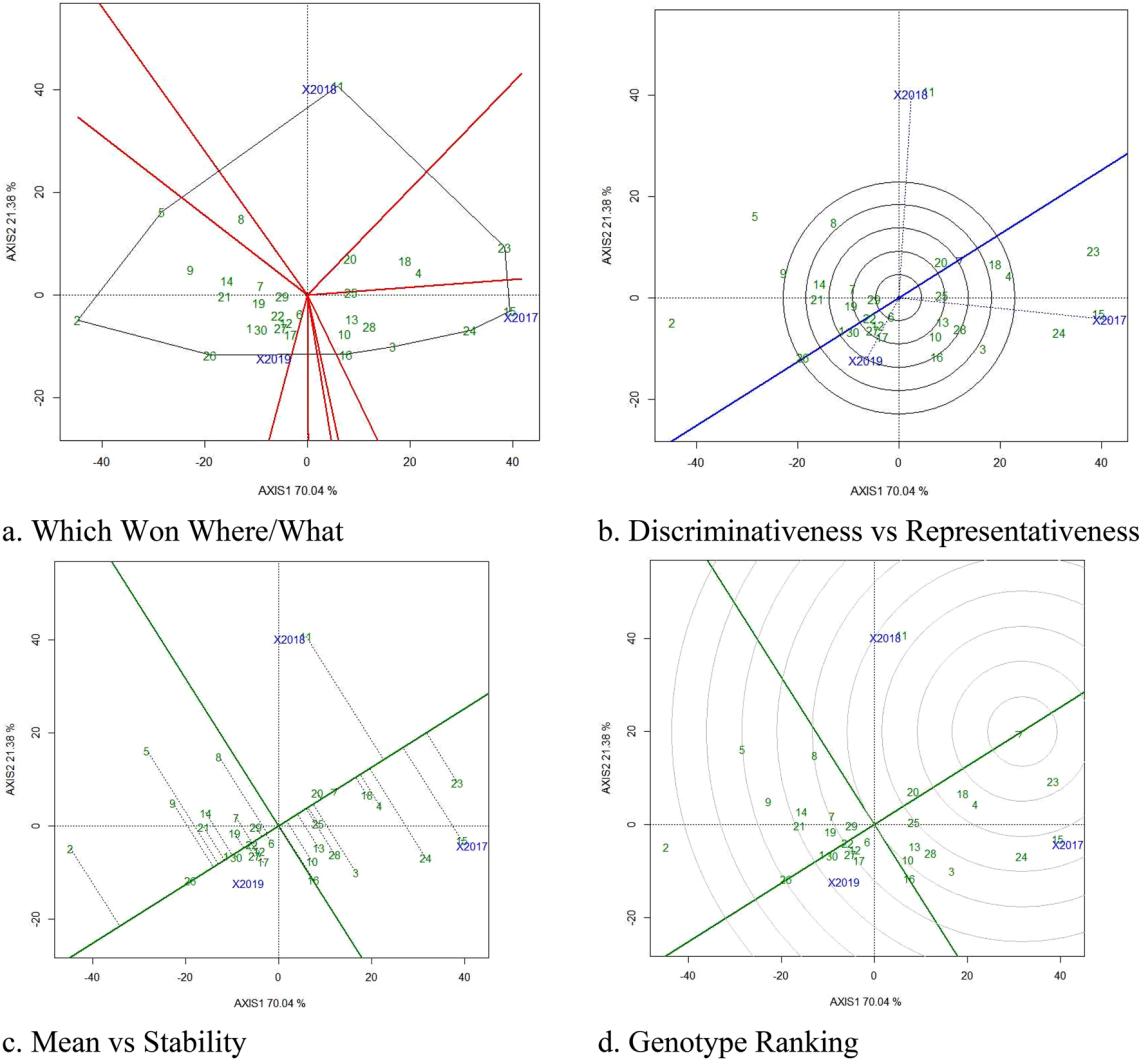
GGE Biplots of for Relative Water Use Efficiency (g/m^2^/mm) under drought stress. The data is not transformed, not scaled, environments were centered (Centering = 2) and the biplots based on singular value partition by column metric preserving. The Biplots explained 79.86 percent of G + GE. (**a**) Which won where/what GGE biplot to visualise the mega environment and environment specific genotypes. (**b**) Discriminativeness vs representativeness GGE biplot depicting the longest environment vector and nearer to average environmental coordinates and identified 2018 as highly discriminative environment, (**c**) mean vs stability GGE biplot depicting the 18 and 23 located in the direction of the average environmental, (**d**) ranking of genotype GGE biplot depicting the genotype 11 located in near to ideal genotype for pod yield (g/m^2^). 1, Abhaya; 2, AK 265; 3, Anantha; 4, CSMG 84-1; 5, Dh3-30; 6, Dharani; 7, DRG 17; 8, DSG 41; 9, GG 2; 10, Girnar 2; 11, GPBD 5; 12, ICGS 1; 13, ICGS 44; 14, ICGS 76; 15, ICGV 86031; 16, ICGV 86325; 17, ICGV 91114; 18, K 6; 19, K 9; 20, Kadiri 5; 21, Kadiri Hariandra; 22, Mutant 3; 23, R 2001-2; 24, R 2001-3; 25, R 8808; 26, Spanish Improved; 27, TAG 24; 28, TDG 39; 29, TG 72; 30, TMV 2.

‘Which Won Where Biplots’ aids in visualising mega environments and identifying superior genotypes for drought environments. These biplots are known as ‘Which Won Where biplots’ because they plot the genotypic mean against the first interaction main components^1,18,32^ to identify winning genotype for each environment. The irregular polygons divide the biplots into the vector and aid in determining the appropriate genotype for each environmental sector. In our investigations, GPBD 5 was the best genotype for pod yield and RUE in 2018, Spanish Improved was the best in 2019, and R 2001-3 was the best genotype in 2017 (Figs. 2a, 3a).

GGE Biplots were utilised to depict the effects of environment, genotype ranking, GEI pattern, and identification of stable environments^33^. The discriminativeness vs. representative perspectives of GGE Biplots aid in identifying the appropriate environments with the most discriminative capacity for genotype differentiation. The type-1, type-2, and type-3 environments can be seen using average environmental coordinates (AEC) and test environments. Type-1 environments are represented by short vectors with average discriminative power, which indicate genotype performance on average. Type-2 environments are depicted as the longest vectors with the best discriminative capacities, capable of discriminating genotype performance. Type-3 environments are indicated by the longest vector with big angles, suited to the negative effects of the environment. The best environments have the longest genotypic vector and are placed on or at acute angles to the AEC^32^. Rainy season of ‘2019’ and ‘2018’ has the longest environmental vector with narrow angles to AEC with the highest discriminative power in our experiments for pod yield and RUE respectively and is regarded the optimal environment to discriminate drought tolerant genotype (Figs. 2b, 3b). The shortest environmental vector ‘2018’ and ‘2019’ detected for pod yield and RUE indicate an average or similar genotype performance.

The mean vs stability biplots aid in understanding genotype mean performance across drought situations. Genotypes that are positioned close or in the direction of AEC are regarded excellent and best performing genotypes^32^. Kadiri 5 was the most stable and better performing variety, for pod yield (Fig. 2c) and RUE (Fig. 3c), with AK 265 and Spanish Improved doing poorly. According to our findings, Kadiri 5 and TG 72, located on or near the AEC, are the most stable and high-performing genotypes, but Dh 3-30 and DSG 41, which are placed away from the AEC, are less stable. Varieties Mutant 3 and TAG 24 were located on AEC axis and hence are highly stable but poor performers for Pod yield and RUE.

The genotype ranking on biplots aids in visualising the ideal genotypes based on their positions in the concentric circle^1,18,32^. None of the genotypes were located in the center of concentric circle however, genotypes Anantha and R 2001-2 were located closer to the center of the concentric circle for pod yield and RUE respectively (Figs. 2d, 3d), indicating its stable performance across environments, whereas AK 265 and Spanish improved were located on the last concentric circle for pod yield and RUE and were considered less stable under drought stress.

## Discussion

Groundnut (*Arachis hypogaea* L.) is one of the world's most widely cultivated food legumes, prized for its high protein and unsaturated oil content while, drought stress is one of the most significant restrictions affecting its production. The Rayalasema region (Anantapur, Kurnool, and Chittoor) of Andhra Pradesh and neighboring districts of Karnataka (Bellary, Chickballapur, Chitradurga, and Tumkur) account for roughly 20–25% of the country’s groundnut area. These areas are in dry zones, where rainfall distribution is variable and drought is a common occurrence. The main crop farmed in these areas under rainfed circumstances is groundnut, which suffers from periodic dry spells. Some groundnut varieties have been identified as drought tolerant^14^, but they have yet to be confirmed in actual drought hot spots. As a result, the goal of this study was to test 30 drought tolerant varieties in Anantapur’s natural drought hot spot conditions during rainy seasons without protective irrigation in order to determine the most stable drought tolerant variety.

The AMMI stability model is commonly used to comprehend the GEI pattern and to find stable cultivars from target environments^23–25,31^. The AMMI ANOVA revealed significant contributions from environments, genotypes, and GEI for pod yield and RUE, which is consistent with prior findings^1,27^. The sum of squares due to GEI_Signal_ is greater than the GEI_Noise_ for pod yield and RUE due to a greater contribution from the additive main effect of genotype and drought stress environments. AMMI model, followed by MASI and SSI stability models, and AMMI 1 biplots, identified Kadiri 5 as the most stable high yielding variety under drought stress conditions for pod yield and RUE, and hence is considered as drought tolerant groundnut variety.

GGE biplots aid in elucidating the interrelationship between environments, rating genotypes, and identifying the best performing genotype in a given environment^27^. According to our findings, the discriminativeness vs representative biplot indicated that ‘2018’ was the ideal year to discriminate genotype for pod yield and RUE because this season experienced severe dry spells from July 23rd to September 3rd, 2018 and October 1st to November 12th, 2018, with rainfall during cropping season being the lowest of the three seasons (Fig. 1S and Table 2S). As a result, the rainy season of ‘2018’ is preferable for identifying drought tolerant genotypes. The genotypes Kadiri 5 was found at an acute angle with an optimal environment for pod yield and RUE and might be considered ideal drought tolerant genotypes for these traits. With the fluctuations in annual weather pattern, Kadiri 5 identified under ideal drought conditions will be able to provide highest yields over the period of time under drought stress conditions.

## Conclusion

Drought significantly impacted varietal performance, as demonstrated by AMMI and GGE stability models in the present study. Rainy season of 2019 facilitated clear differentiation of drought-tolerant varieties identifying Kadiri 5 as the most suitable cultivars for drought-prone regions.

### Supplementary Information

Supplementary Information.

## Data Availability

All relevant data are within the manuscript and in Supporting Information files.

## Abbreviations

AEC: Average environmental coordinates
AMMI: Additive main effect and multiplicative interaction
DAS: Days after sowing
GEI: Genotype*Environment Interactions
GGE: Genotype-genotype*Environment Interaction
MASI: Modified AMMI Stability Index
RUE: Rainfall-use-efficiency
RCBD: Randomised complete block design
SSI: Simultaneous stability index
