# Supplementary material for: Integrating data from asymmetric multi-models can identify drought-resistant groundnut genotypes for drought hot-spot locations
Source: Sci Rep. 2023 Aug 5;13:12705. doi: 10.1038/s41598-023-38581-0 (PMC10404259; doi:10.1038/s41598-023-38581-0)
Supplement: Supplementary file 1 — Supplementary Information. [file 41598_2023_38581_MOESM1_ESM.docx]

**Table 1S: All India area, production and productivity during Kharif season for the years 2017-18 to 2019-20**

| States | Area (Hectare) | | | Production (Tonnes) | | | | Yield (Tonnes/Hectare) | | | |
| --- | --- | --- | --- | --- | --- | --- | --- | --- | --- | --- | --- |
|  | 2017-18 | 2018-19 | 2019-20 | 2017-18 | 2018-19 | 2019-20 | 2017-18 | | 2018-19 | 2019-20 |  |
| Andhra Pradesh | 647930 | 687139 | 567875 | 836554 | 332910 | 621906 | 1.29 | | 0.48 | 1.10 |  |
| Arunachal Pradesh | 919 | 921 | 923 | 901 | 903 | 905 | 0.98 | | 0.98 | 0.98 |  |
| Bihar | 569 | 856 | 841 | 580 | 873 | 858 | 1.02 | | 1.02 | 1.02 |  |
| Chhattisgarh | 25406 | 23945 |  | 32112 | 27190 |  | 1.26 | | 1.14 |  |  |
| Goa | 280 | 210 | 2 | 651 | 498 | 5 | 2.33 | | 2.37 | 2.50 |  |
| Gujarat | 1627275 | 1566372 | 1629275 | 3970876 | 2143341 | 4503628 | 2.44 | | 1.37 | 2.76 |  |
| Haryana | 3100 | 3490 | 2725 | 3600 | 3993 | 3089 | 1.16 | | 1.14 | 1.13 |  |
| Himachal Pradesh | 36 | 42 | 44 | 37 | 43 | 51 | 1.03 | | 1.02 | 1.16 |  |
| Jharkhand | 32273 | 30072 | 26088 | 35843 | 31046 | 28782 | 1.11 | | 1.03 | 1.10 |  |
| Karnataka | 389994 | 397745 | 413854 | 429732 | 271955 | 369876 | 1.10 | | 0.68 | 0.89 |  |
| Madhya Pradesh | 218160 | 215697 | 217499 | 338130 | 398873 | 345171 | 1.55 | | 1.85 | 1.59 |  |
| Maharashtra | 239847 | 217336 | 221102 | 276066 | 203522 | 191432 | 1.15 | | 0.94 | 0.87 |  |
| Manipur | 3340 | 3216 |  | 3142 | 2995 |  | 0.94 | | 0.93 |  |  |
| Nagaland | 970 | 1010 | 1050 | 1010 | 1050 | 1100 | 1.04 | | 1.04 | 1.05 |  |
| Puducherry | 2 | 3 | 1 | 6 | 9 | 3 | 3.00 | | 3.00 | 3.00 |  |
| Punjab | 1200 | 1300 | 1400 | 2300 | 2600 | 2800 | 1.92 | | 2.00 | 2.00 |  |
| Rajasthan | 628516 | 673366 | 739022 | 1262214 | 1382866 | 1623352 | 2.01 | | 2.05 | 2.20 |  |
| Tamil Nadu | 188544 | 196543 | 209159 | 463781 | 426219 | 518418 | 2.46 | | 2.17 | 2.48 |  |
| Telangana | 21006 | 13157 | 12363 | 45965 | 32947 | 33907 | 2.19 | | 2.50 | 2.74 |  |
| UT’s | 17 | 3 | 3 | 16 | 5 | 4.5 | 0.94 | | 1.67 | 1.50 |  |
| Tripura | 1121 | 1257 | 1207 | 1343 | 1669 | 1613 | 1.20 | | 1.33 | 1.34 |  |
| Uttar Pradesh | 87922 | 101032 | 93822 | 89075 | 100470 | 88371 | 1.01 | | 0.99 | 0.94 |  |
| Uttarakhand | 805 | 709 | 669 | 1109 | 735 | 706 | 1.38 | | 1.04 | 1.06 |  |
| Total | 4119232 | 4135421 | 4138924 | 7795043 | 5366713 | 8335978 | 1.89 | | 1.30 | 2.01 |  |

**Table 2S: Performance of groundnut genotypes for pod yield and Rain water use efficiency during 2017 to 2019**

| S. No. |  | Pod yield (g/m2) | | | Rain water use efficiency (mg/m^2^/mm) | | |
| --- | --- | --- | --- | --- | --- | --- | --- |
|  | Genotype | 2017 | 2018 | 2019 | 2017 | 2018 | 2019 |
| 1 | Abhaya | 288.0 h-k | 9.3 b-c | 178.1 e-i | 57.83 h-k | 4.45 i-j | 33.73 e-i |
| 2 | AK 265 | 130.5 n | 16.1 b-c | 264.8 a-b | 26.21 n | 7.64 g-j | 50.14 a-b |
| 3 | Kadiri Anantha | 435.0 b | 13.2 b-c | 222.9 b-e | 87.35 b-c | 6.29 h-j | 42.22 a-e |
| 4 | CSMG 84-1 | 449.5 b | 40.1 b-c | 176.1 e-j | 90.25 b | 19.07 b-f | 33.36 e-i |
| 5 | Dh 3-30 | 197.0 m | 57.1 b-c | 194.3 e-g | 39.56 m | 27.20 b-c | 36.79 d-f |
| 6 | Dharani | 329.1 g-h | 10.9 b-c | 127.7 j-k | 66.09 f-i | 5.20 h-j | 24.19 i |
| 7 | DRG 17 | 300.0 h-k | 31.6 b-c | 209.6 c-g | 60.24 g-k | 15.06 e-i | 39.70 b-f |
| 8 | DSG 41 | 278.2 i-l | 60.5 b | 216.5 b-f | 55.86 i-l | 28.79 b | 41.01 b-f |
| 9 | GG 2 | 232.4 l-m | 37.1 b-c | 221.4 b-e | 46.66 l-m | 17.64 c-g | 41.93 a-e |
| 10 | Girnar 2 | 378.1 d-f | 8.4 b-c | 159.6 g-k | 75.93 d-f | 4.01 j | 30.23 f-i |
| 11 | GPBD 5 | 352.3 e-g | 109.5 a | 122.5 k | 70.75 e-g | 52.14 a | 23.21 i |
| 12 | ICGS 1 | 333.0 f-h | 23.1 b-c | 258.5 a-c | 66.87 f-h | 11.00 f-j | 48.96 a-c |
| 13 | ICGS 44 | 386.3 c-e | 16.7 b-c | 172.3 e-k | 77.56 c-e | 7.93 g-j | 32.62 e-i |
| 14 | ICGS 76 | 267.5 j-l | 33.0 b-c | 213.1 c-f | 53.70 j-l | 15.71 d-h | 40.36 b-f |
| 15 | ICGV 86031 | 546.0 a | 32.8 b-c | 217.5 b-f | 109.64 a | 15.61 d-h | 41.19 b-f |
| 16 | ICGV 86325 | 387.6 c-e | 6.6 c | 213.8 c-f | 77.83 c-e | 3.15 j | 40.48 b-f |
| 17 | ICGV 91114 | 337.0 f-h | 16.7 b-c | 246.3 a-d | 67.66 e-h | 7.96 g-j | 46.64 a-d |
| 18 | K 6 | 429.0 b-c | 38.1 b-c | 126.9 j-k | 86.14 b-d | 18.16 c-g | 24.03 i |
| 19 | K 9 | 296.0 h-k | 21.6 b-c | 188.6 e-g | 59.44 h-k | 10.28 f-j | 35.73 e-h |
| 20 | Kadiri 5 | 384.6 c-e | 46.4 b-c | 201.9 d-g | 77.23 c-e | 22.11 b-e | 38.23 d-f |
| 21 | Kadiri Harithandra | 262.5 k-l | 22.4 b-c | 184.8 e-h | 52.71 k-l | 10.68 f-j | 35.00 e-h |
| 22 | Mutant 3 | 315.0 g-j | 16.6 b-c | 183.3 e-i | 63.25 g-k | 7.90 g-j | 34.72 e-h |
| 23 | R 2001-2 | 531.0 a | 53.6 b-c | 167.8 f-k | 106.63 a | 25.54 b-d | 31.78 e-i |
| 24 | R 2001-3 | 504.0 a | 18.6 b-c | 183.8 e-i | 101.20 a | 8.85 f-j | 34.81 e-h |
| 25 | R 8808 | 378.7 d-f | 23.6 b-c | 135.9 h-k | 76.05 d-f | 11.24 f-j | 25.74 g-i |
| 26 | Spanish Improved | 263.0 k-l | 8.1 b-c | 274.4 a | 52.82 k-l | 3.84 j | 51.97 a |
| 27 | TAG 24 | 319.5 g-i | 12.3 b-c | 191.4 e-g | 64.16 g-j | 5.85 h-j | 36.25 d-g |
| 28 | TDG 39 | 405.6 b-d | 16.0 b-c | 185.4 e-h | 81.45 b-d | 7.61 g-j | 35.12 e-h |
| 29 | TG 72 | 312.0 g-k | 18.5 b-c | 133.7 i-k | 62.65 g-k | 8.79 f-j | 25.32 h-i |
| 30 | TMV 2 | 302.1 h-k | 12.8 b-c | 206.9 d-g | 60.66 g-k | 6.07 h-j | 39.18 c-f |
|  | **Average** | **344.3** | **27.7** | **192.7** | **69.14** | **13.19** | **36.49** |

Fig 1S: Maximum (Tmax °C) and Minimum (Tmin °C) temperature and rainfall (mm) recorded during Rainy seasons of 2017, 2018 and 2019
